# Investigation of the Relationship Between Tumor Microenvironment and Prognostic Parameters in Invasive Breast Carcinomas of No Special Type: A Retrospective Analysis

**DOI:** 10.5146/tjpath.2024.12805

**Published:** 2024-09-02

**Authors:** Mine Ozsen, Sahsine Tolunay, Kazım Senol, Adem Deligonul, Sehsuvar Gokgoz, Turkkan Evrensel

**Affiliations:** Department of Pathology, Bursa Uludag University, Faculty of Medicine, Bursa, Türkiye; Department of General Surgery, Bursa Uludag University, Faculty of Medicine, Bursa, Türkiye; Department of Medical Oncology, Bursa Uludag University, Faculty of Medicine, Bursa, Türkiye

**Keywords:** Breast cancer, Tumor budding, Tumor infiltrating lymphocytes, Tumor microenvironment, Prognosis

## Abstract

***Objective: *** The tumor microenvironment is a heterogeneous and constantly changing territory that plays an active role in tumor formation and progression. It constantly interacts with tumor cells, plays an active role in tumor development, and even appears as a parameter of prognostic importance, and the importance of the tumor microenvironment in breast cancer has been emphasized by recent studies. In this study, we aimed to retrospectively evaluate the relationship between the tumor microenvironment and prognostic parameters in invasive breast carcinomas of no special type.

***Material and Methods:*** A total of 271 cases diagnosed as invasive breast carcinoma of no special type from resection materials in our center between 2007 and 2015 were included in the study. Hematoxylin-eosin stained slides with a thickness of 4-5 micrometers were evaluated in terms of tumor infiltrating lymphocytes, peritumoral and intratumoral desmoplastic reaction, intratumoral and peritumoral tumor budding, stromal features, and tumor growth pattern.

***Results: *** When parameters related to the tumor microenvironment were compared with other prognostic parameters, there was a significant relationship between TILs and tumor grade, size, stage, immunohistochemical subgroup and Ki-67 proliferation index. A significant relationship was detected between intratumoral stromal reaction and tumor grade, size, molecular subgroup and the Ki-67 proliferation index (p<0.05). When stroma and other prognostic parameters were compared, tumors with desmoplastic stroma had higher grades and higher Ki-67 proliferation indexes, and they were observed more frequently in the triple negative molecular subgroup.

***Conclusion:*** We believe that including parameters related to tumor microenvironment in breast cancer reports, which hold a prognostic and predictive importance, will contribute to patient management. Considering the fact that these can be easily evaluated from routinely used hematoxylin-eosin stained slides, this does not cause additional costs or excessive time loss.

## Introduction

Over the years, research has been conducted to reveal the relationship between the prognosis and various histopathological (invasive tumor size, histopathological type, grade, lymphovascular invasion, and axillary lymph node status), immunohistochemical (estrogen receptor, progesterone receptor, c-erbB2 status, and ki-67 proliferative index) and molecular (HER2 status) parameters in breast cancer (1,2).

Another parameter whose impact on the prognosis has been investigated is the tumor microenvironment. The tumor microenvironment can be defined as a heterogeneous and constantly changing territory that plays an active role in tumor formation and progression. Studies have shown that the tumor microenvironment, consisting of tumor cells and various non-neoplastic cells (fibroblasts, immune cells, endothelial cells, inflammatory cells, adipocytes, signaling molecules, extracellular matrix components) plays a role in the emergence, development and treatment response of breast cancer (3). Therefore, we aimed to investigate the relationship between the tumor microenvironment and prognostic parameters in invasive breast carcinoma of no special type (IBC-NST) cases that had not undergone any neoadjuvant treatment.

## Materials and Method

All cases diagnosed with IBC-NST from resection materials (lumpectomy, segmental mastectomy, modified radical mastectomy, breast-conserving surgical material) in our center, between 2007 and 2015, were included in the study. Cases where the slides were not available or not suitable for re-evaluation, cases who received neoadjuvant treatment, cases diagnosed from non-resection materials, and cases histologically diagnosed as other than IBC-NST were excluded from the study.

Demographic (age) and clinicopathological (tumor site, size, grade, presence of lymphovascular invasion, perineural invasion, immunohistochemical subgroups (according to immunohistochemical staining results; ER positive and low ki-67 proliferation index, ER positive and high ki-67 proliferation index, HER2 positive and triple negative), presence of metastasis, site of metastases if present, stage, presence of recurrence, survival status, recurrence-free, total survival and follow-up periods) of the cases were obtained from patient files and pathology reports.

Hematoxylin-eosin (H&E) stained slides with a thickness of 4-5 micrometers included in the study were evaluated in terms of parameters related to the tumor microenvironment (tumor infiltrating lymphocytes (TILs), peritumoral and intratumoral desmoplastic reaction, intratumoral and peritumoral tumor budding, stromal features and tumor growth pattern).

Two different methods were used when evaluating the TIL. The first was based on the evaluation technique recommended by the World Health Organization (WHO) Breast Tumors Classification in 2019. Accordingly, the ratio of mononuclear inflammatory cells to the stromal area in the intratumoral compartment was taken as the basis. The entire tumor area was evaluated and a net percentage value was obtained for each tumor from a single slide. The percentage values were grouped as <10%, between 10% and 50%, and >50%, based on the recommended threshold values (1,4,5). The second evaluation method included evaluating the distribution of mononuclear inflammatory cells in the tumor stroma. Tumors were divided into two groups as diffuse and non-diffuse TIL.

When evaluating the peritumoral desmoplastic reaction, the stromal area around the tumor was considered. Desmoplasia was categorized as absent, mild, moderate and prominent. Similar grading was used for the evaluation of the intratumoral desmoplastic reaction, except for including the stromal area within the tumor and not the tumor periphery.

Tumor budding was defined as isolated tumor cells or groups of less than 5 tumor cells. Tumor budding was noted as present or absent. It was evaluated from hotspots of two separate sites, using 400x magnification. One site was the border between invasive tumor and the surrounding stroma (peritumoral), and the other was within the invasive tumor (intratumoral) (6).

Stromal features were evaluated and recorded, and the stroma was divided into three categories as myxoid, desmoplastic and hyalinized.

Growth patterns were assessed based on publications that report growth patterns to be associated with the prognosis in metastatic liver tumors. Accordingly, tumor growth patterns were defined as “desmoplastic” if there was a desmoplastic rim between the tumor cells and the surrounding stroma, “replacement” if tumor cells were observed between normal structures without destroying the main architecture, and “pushing” if tumor cells were growing by pushing the normal structures (7).

Approval for the research, dated 25 November 2020 and numbered 2020-21/10, was obtained from the local Clinical Research Ethics Committee.

### Statistical Analysis

The SPSS 25.0 package program was used for statistical analyses. Categorical measurements were given as numbers and percentages. Continuous measurements were calculated as mean and standard deviation (median and minimum-maximum where appropriate). The chi-square test or Fisher’s exact test was used to compare categorical variables. The distribution of the groups was checked for comparison of continuous measurements. Student’s T test was used for parameters with a normal distribution. Anova was used for the comparison of more than two variables. The Mann-Whitney U test was used for parameters that did not show a normal distribution and the Kruskal-Wallis test was used for the comparison of more than two variables. The Kaplan-Meier method was used to evaluate the survival curve and the Long-rank test was used to calculate the difference of survival between groups. A statistical significance level of 0.05 was determined in all tests.

## Results

A total of 271 cases diagnosed with IBC-NST from resection materials in our center, between 2007 and 2015, and which met the study criteria were identified. The general characteristics of the cases and the routine parameters evaluated in resection materials are summarized in Table 1 and Table 2 (Table 3, Table 4 and Table 5).

**Table 1:** General characteristics of the cases (n=271)

	**Mean + SD**	**Min-Max**
Age (years)	54.8±12.1	27-83
Size (cm)	2.6±1.5	0.3-10
Follow-up period	69.6±33.9	1-156
Recurrence-free survival (months)	88.3±30.3	3-156
Total survival (months)	91.7±28.1	3-156
	**n (%)**	
Stage 1 2 3 4	78 (28.8) 144 (53.1) 38 (14) 11 (4.1)	
Recurrence Absent Present	251 (92.6) 20 (7.4)	
Status Dead of disease Alive	59 (21.8) 212 (78.2)	

**Table 2:** Histopathological features of the cases (n=271)

	**n (%)**
TILs <10% 10-50% >50%	183 (67.5) 44 (16.3) 44 (16.2)
TILs Diffuse Non-diffuse	193 (71.2) 78 (28.8)
Peritumoral stromal reaction Absent Mild Moderate Prominent	196 (72.3) 65 (24) 3 (1.1) 7 (2.6)
Intratumoral stromal reaction Absent Mild Moderate Prominent	3 (1.1) 50 (18.5) 56 (20.7) 162 (59.8)
Peritumoral tumor budding Absent Present	98 (36.2) 173 (63.8)
Intratumoral tumor budding Absent Present	78 (28.8) 193 (71.2)
Stroma Myxoid Desmoplastic Hyalinized	67 (24.7) 91 (33.6) 113 (41.7)
Growth pattern Replacement Pushing Desmoplastic	244 (90) 19 (7) 8 (3)
Grade 1 2 3	9 (3.3) 115 (42.4) 147 (54.2)
Lymphovascular invasion Absent Present	197 (72.7) 74 (27.3)
Perineural invasion Absent Present	198 (73.1) 73 (26.9)
Immunohistochemical subgroup ER positive HER-2 positive Triple negative	187 (69) 19 (7) 65 (24)
Ki-67 percentage ≤14% >14%	77 (28.4) 194 (71.6)
Axillary metastasis Absent Present	147 (54.2) 124 (45.8)

**Table 3:** Relation of Tumor Microenvironment to Prognostic Parameters (n=271)

**TIL**												
		**≤10 n (%)**				**10-50 n (%)**		**≥50 n (%)**				**p**
**Grade**	1	8	4.4			1	2.3	0			0.0	**0.0001**
	2	93	50.8			12	27.3	10			22.7	
	3	82	44.8			31	70.5	34			77.3	
**Lvi**	Absent	134	73.2			28	63.6	35			79.5	0.236
	Present	49	26.8			16	36.4	9			20.5	
**Perineural Invasion**	Absent	129	70.5			33		36			81.8	0.299
	Present	54	29.5			11	25.0	8			18.2	
**Immunohistochemical Groups**	ER positive	150	82.0			26	59.1	11			25.0	**0.0001**
	HER2 positive	8	4.4			4	9.1	7			15.9	
	Triple Negative	25	13.7			14	31.8	26			59.1	
**Ki-67 (%)**	≤14	65	35.5			7	15.9	5			11.4	**0.001**
	>14	118	64.5			37	84.1	39			88.6	
**Axillary Metastasis**	Absent	94	51.4			24	54.5	29			65.9	0.220
	Present	89	48.6			20	45.5	15			34.1	
**Stage**	1	59	32.2			8	18.2	11			25.0	**0.030**
	2	101	55.2			21	47.7	22			50.0	
	3	18	9.8			12	27.3	8			18.2	
	4	5	2.7			3	6.8	3			6.8	
**Presence of Recurrence**	Absent	166	90.7			43	97.7	42			95.5	0.205
	Present	17	9.3			1	2.3	2			4.5	
**Survival Status**	Exitus	141	77.0			35	79.5	36			81.8	0.768
	Alive	42	23.0			9	20.5	8			18.2	
**Intratumoral stromal reaction**												
		**Absent n (%)**				**Mild n (%)**		**Moderate n (%)**				**p**
**Grade**	1	0	0.0			1	1.8	8			4.9	**0.001**
	2	11	20.8			26	46.4	78			48.1	
	3	42	79.2			29	51.8	76			46.9	
**Lvi**	Absent	42	79.2			41	73.2	114			70.4	0.451
	Present	11	20.8			15	26.8	48			29.6	
**Perineural Invasion**	Absent	44	83.0			38	67.9	116			71.6	0.164
	Present	9	17.0			18	32.1	46			28.4	
**Immunohistochemical Groups**	ER positive	20	37.7			39	69.6	128			79.0	**0.0001**
	HER2 positive	5	9.4			6	10.7	8			4.9	0.451
	Triple Negative	28	52.8			11	19.6	26			16.0	
**Ki-67 (%)**	≤14	4	7.5			16	28.6	57			35.2	**0.001**
	>14	49	92.5			40	71.4	105			64.8	
**Axillary Metastasis**	Absent	33	62.3			30	53.6	84			51.9	0.415
	Present	20	37.7			26	46.4	78			48.1	0.451
**Stage**	1	14	26.4			18	32.1	46			28.4	0.419
	2	26	49.1			26	46.4	92			56.8	
	3	10	18.9			11	19.6	17			10.5	
	4	3	5.7			1	1.8	7			4.3	0.415
**Presence of Recurrence**	Absent	49	92.5			51	91.1	151			93.2	0.869
	Present	4	7.5			5	8.9	11			6.8	0.451
**Survival Status**	Exitus	41	77.4			40	71.4	131			80.9	0.768
	Alive	12	22.6			16	28.6	31			19.1	
**Intratumoral tumor budding**												
		**Absent n (%)**					**Present n (%)**					**p**
**Immunohistochemical Groups**	ER positive	42			53.8		145			75.1		0.0001
	HER2 positive	12			15.4		7			3.6		
	Triple Negative	24			30.8		41			21.2		
**Axillary Metastasis**	Absent	51			65.4		96			49.7		**0.022**
	Present	27			34.6		97			50.3		
**Peritumoral tumor budding**												
		**Absent n (%)**					**Present n (%)**					**p**
**Lvi**	Absent	79			80.6		118			68.2		**0.033**
	Present	19			19.4		55			31.8		
**Stage**	1	42			42.9		102			59.0		0.023
	2	21			21.4		17			9.8		
	3	4			4.1		7			4.0		
	4	31			31.6		47			27.2		
**Stroma**												
		**Myxoid n (%)**				**Desmoplastic n (%)**		**Hyalinized n (%)**				**p**
**Lateralisation**	Left	31	46.3			34	37.4	65			57.5	**0.016**
	Right	36	53.7			57	62.6	48			42.5	
**Grade**	1	3	4.5			1	1.1	5			4.4	**0.003**
	2	40	59.7			29	31.9	46			40.7	
	3	24	35.8			61	67.0	62			54.9	
**Immunohistochemical Groups**	ER positive	61	91.0			53	58.2	73			64.6	**0.0001**
	HER2 positive	3	4.5			8	8.8	8			7.1	
	Triple Negative	3	4.5			30	33.0	32			28.3	
**Ki-67 (%)**		38	56.7			13	14.3	26			23.0	**0.0001**
		29	43.3			78	85.7	87			77.0	**0.0001**
**Stage**	1	5	7.5			33	36.3	40			35.4	**0.001**
	2	48	71.6			43	47.3	53			46.9	
	3	13	19.4			10	11.0	15			13.3	
	4	1	1.5			5	5.5	5			4.4	
**Growth Pattern**												
		**Desmoplastic n (%)**				**Replacement n (%)**			**Pushing n (%)**			**p**
**Grade**	1	0		0.0		9	3.7		0	0.0		**0.028**
	2	3		37.5		110	45.1		2	10.5		
	3	5		62.5		125	51.2		17	89.5		
**Immunohistochemical Groups**	ER positive	6		75.0		177	72.5		4	21.1		**0.0001**
	HER2 positive	1		12.5		18	7.4		0	0.0		
	Triple Negative	1		12.5		49	20.1		15	78.9		
**Ki-67 (%)**	≤14	2		25.0		75	30.7		0	0.0		**0.016**
	>14	6		75.0		169	69.3		19	100.0		
**Axillary Metastasis**	Absent	4		50.0		127			16	84.2		**0.025**
	Present	4		50.0		117	48.0		3	15.8		

**Table 4:** Relationship of tumor microenvironment-related parameters with recurrence-free survival rates

	**Estimated Meana**	**Std. Error**	**95% Confidence Interval**		**5-year recurrence-free survival %**	**p**
			**Lower Bound**	**Upper Bound**		
Recurrence-free Survival	146.8	2.0	142.9	150.7	93.8	-
**TIL (%)**						
≤10	144.2	2.7	138.9	149.6	91.9	0.202
10-50	117.9	2.1	113.9	121.9	97.6	
≥50	129.2	2.6	124.1	134.4	97.6	
**TIL**						
Diffuse	145.7	2.4	140.8	150.6	92.9	0.384
Non-diffuse	141.9	2.9	136.1	147.8	94.4	
**Intratumoral Stromal Reaction**						
Absent - Mild	126.1	3.3	119.6	132.7	93.7	0.881
Moderate	122.8	4.0	114.9	130.7	90.7	
Severe	147.4	2.5	142.5	152.4	93.4	
**Intratumoral Tumor Budding**						
Absent	137.8	3.3	131.3	144.2	93.0	0.903
Present	146.7	2.4	142.0	151.4	93.5	
**Peritumoral Tumor Budding**						
Absent	136.8	3.1	130.7	143.0	93.3	0.661
Present	147.2	2.4	142.4	152.1	94.0	
**Stroma**						
Myxoid	110.7	2.4	105.9	115.5	95.3	0.132
Desmoplastic	150.7	2.6	145.7	155.8	96.2	
Hyalinized	133.4	3.3	126.8	139.9	90.1	
**Growth Pattern**						
Desmoplastic	-	-	-	-	100	0.278
Replacement	147.3	2.1	143.2	151.3	94.4	
Pushing	110.5	7.2	96.4	124.6	83.3	

**Table 5:** Relationship of tumor microenvironment-related parameters with total survival rate

	**Estimated Mean^a^**	**Std. Error**	**95% Confidence Interval**		**1-year survival %**	**3-year survival %**	**5-year survival %**	**p**
			**Lower Bound**	**Upper Bound**				
Total Survival	126.2	3.9	118.4	133.9	97.8	93.4	88.2	-
**TIL (%)**								
≤10	126.1	4.7	116.7	135.3	97.8	91.3	86.9	0.780
10-50	105.3	4.7	95.9	114.6	95.5	90.9	84.0	
≥50	117.5	5.1	107.5	127.5	95.5	93.2	88.5	
**TIL**								
Diffuse	125.3	4.9	115.6	134.9	98.4	92.2	87.6	0.457
Non-diffuse	121.8	6.3	109.5	134.1	94.9	88.5	87.2	
**Peritumoral Stromal Reaction**								
Absent	126.134	5.086	116.165	136.103	98.0	92.9	88.3	0.977
Mild	114.453	4.568	105.500	123.405	96.9	90.8	87.7	
Moderate	120.000	0.000	120.000	120.000	100	100	100	
Severe	99.286	20.270	59.556	139.016	85.7	71.4	71.4	
**Intratumoral Stromal Reaction**								
Absent - Mild	112.9	5.3	102.6	123.3	94.3	90.6	88.6	0.331
Moderate	109.9	5.1	99.9	119.9	98.2	92.9	93.9	
Severe	132.1	4.3	123.7	140.5	98.1	92.6	87.7	
**Intratumoral Tumor Budding**								
Absent	127.1	4.8	117.6	136.4	96.2	92.3	89.7	0.235
Present	120.9	5.3	110.4	131.3	97.9	92.2	86.5	
**Periumoral Tumor Budding**								
Absent	120.8	4.7	111.6	129.9	95.9	91.8	86.7	0.875
Present	124.7	5.8	113.3	136.1	98.8	91.9	88.4	
**Stroma**								
Myxoid	99.5	3.8	91.8	107.1	95.5	91.0	82.1	0.288
Desmoplastic	122.0	6.2	109.8	134.3	95.6	87.9	84.6	
Hyalinized	126.7	3.9	118.9	134.5	99.1	96.5	92.0	
**Growth Pattern**								
Desmoplastic	110.3	15.4	80.1	140.5	87.5	87.5	72.9	0.987
Replacement	124.9	4.7	115.6	134.2	98.0	92.2	87.3	
Pushing	116.0	6.9	102.3	129.7	94.7	89.2	89.2	

**Figure 1:**
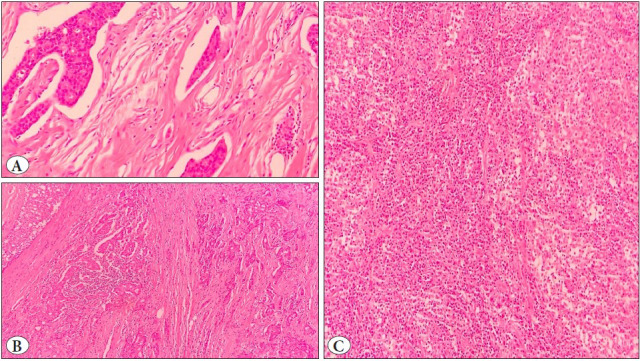
Assessment of tumor-infiltrating lymphocytes in the breast cancer microenvironment. High magnification view of one stromal area included in the scoring, estimated as <10% sTILs (**A,** H&Ex400). High magnification view of one stromal area included in the scoring, estimated as 10-50% sTILs (**B,** H&Ex200). High magnification view of one stromal area included in the scoring, estimated as >50% sTILs (H&Ex400).

The rate of TILs was less than 10% in 183 (67.5%), between 10% and 50% in 44 (16.2%) and more than 50% in 44 (16.2%) cases. 193 (71.2%) tumors showed diffuse TILs and 78 (28.8%) showed non-diffuse TILs (Figure 1).

**Figure 2:**
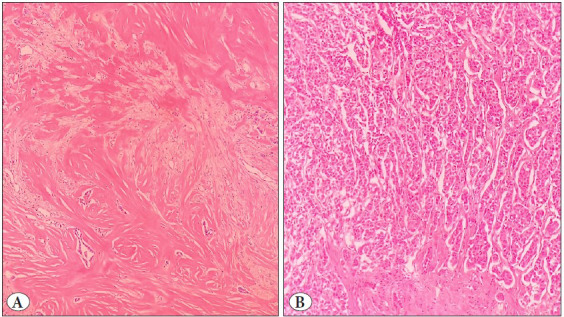
Assessment of intratumoral stromal reaction in the breast cancer microenvironment. Areas with evaluation of intense intratumoral stromal reaction on high magnification view (**A,** H&Ex400). Areas with evaluation of low intratumoral stromal reaction on high magnification view (**B,** H&Ex400).

Peritumoral stromal reaction was absent in 196 (72.3%), mild in 65 (24%), moderate in 3 (1.1%), and prominent in 7 (2.6%) tumors. Intratumoral stromal reaction was absent in 3 (1.1%), mild in 50 (18.5%), moderate in 56 (20.7%), and prominent in 162 (59.8%) tumors (Figure 2).

**Figure 3:**
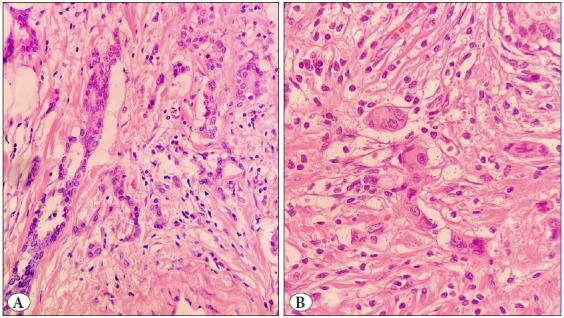
Assessment of tumor budding in the breast cancer microenvironment (**A,B,** H&Ex400).

Peritumoral tumor budding was not detected in 98 (36.2%), and was present in 173 (63.8%) cases. Intratumoral tumor budding was absent in 78 (28.8%) and present in 193 (71.2%) tumors (Figure 3).

Stroma was myxoid in 67 (24.7%), desmoplastic in 91 (33.6%), and hyalinized in 113 (41.7%) tumors.

A replacement growth pattern was observed in 90% of the tumors, while a pushing pattern and desmoplastic pattern was present in 7% and 3%, respectively.

### Comparison of TILs and Other Prognostic Parameters

When features related to the tumor microenvironment were compared with other prognostic parameters, a statistically significant relationship was found between TILs and grade, tumor size, molecular subgroup, ki-67 percentage, and tumor stage. Accordingly, the rate of TILs was higher in tumors with a larger size, higher grade, and a higher ki-67 proliferation index. TILs and size, grade, and ki-67 percentage were directly proportional (p=0.014, p=0.0001, p=0.001). In addition, an inverse relationship was found between TILs and tumor stage. As the rate of TILs decreased, the tumor stage increased (p=0.030). The rate of TILs was significantly higher in the Her2 and triple negative molecular subgroups compared to the luminal subgroup (p=0.0001). When TILs were categorized as diffuse and non-diffuse, significant statistical results could not be obtained.

### Comparison of Intratumoral Stromal Reaction and Other Prognostic Parameters

A statistically significant relationship was present between intratumoral stromal reaction intensity and tumor grade, size, molecular subgroup, and ki-67 proliferation index. Grade, tumor size and ki-67 proliferation index increased as the intensity of intratumoral stromal reaction decreased (p=0.007, p=0.001, p=0.001). The stromal reaction intensity in Her2 and triple negative molecular subgroups was lower than in the luminal subgroup (p=0.0001).

### Comparison of Tumor Budding and Other Prognostic Parameters

Incidence of axillary metastasis was higher in cases showing intratumoral tumor budding. The lymphovascular invasion incidence was higher in cases showing peritumoral tumor budding (p=0.022, p=0.033).

### Comparison of Stromal Features and Other Prognostic Parameters

Tumors with a desmoplastic stroma were higher grade tumors, the ki-67 proliferation indices were higher, and were most frequently in the triple negative subgroup (p=0.003, p=0.0001 and p=0.0001). Tumors with a myxoid stroma were predominantly in the luminal subgroup, whereas tumors with a hyalinized stroma had a higher ki-67 proliferation index compared to tumors with a myxoid stroma (p=0.003, p=0.0001).

### Comparison of Growth Pattern and Other Prognostic Parameters

In cases with axillary metastases, a desmoplastic growth pattern was more frequent and the pushing growth pattern was seen at a lower rate (p=0.025).

### Comparison of Peritumoral Stromal Reaction and Other Prognostic Parameters

A statistically significant correlation was not detected between peritumoral stromal reaction intensity and other prognostic parameters.

### Relationship Between Tumor Microenvironment and Survival

The mean follow-up period was 69.6±33.9 months. Recurrence-free survival and total survival periods ranged from 3 to 156 months. Mean recurrence-free survival period was 88.3±30.3 months. Mean total survival period was 91.7±28.1 months. A total of 59 patients died during the follow-up period. The 1, 3, and 5-year recurrence-free survival rates were 98.9%, 95.8%, and 93.8%, while overall survival rates were 97.8%, 93.4%, and 88.2%, respectively. Recurrence-free and total survival periods were not statistically related to the parameters of the tumor microenvironment (p>0.05).

## Discussion

The tumor microenvironment is constantly interacting with tumor cells, plays an active role in tumor development and even provides prognostic information in some tumors. It is formed as a result of genetic changes in the tumor cells. The tumor microenvironment is observed differently in every tumor type and is tumor-specific. It has been investigated thoroughly in malignant melanoma and lung and colorectal carcinomas where immune cell infiltration is more frequent (8–10). Although immune cell infiltration is less common in breast cancer, it has been reported that the tumor microenvironment is effective in tumor development, progression, and treatment response (11,12). After its first report in 1922, the assessment of TILs was included in the 5th edition of the WHO classification of breast tumors, published in 2019. Accordingly, the evaluation is based on the ratio of mononuclear inflammatory cells to the stromal area, in the intratumoral stromal compartment. The evaluation should include the entire tumor area and should be done on a 20x or 40x objective on a single slide (1,13). Various studies on non-mammary tumors show that tumors with high TILs rates have a better prognosis compared to tumors with low TILs rates (10). The same results were obtained from similar studies on breast carcinomas. Accordingly, higher TILs rates are associated with better prognosis and better treatment responses, especially in HER2 and triple-negative tumors, known to have poorer prognoses (1,3,12,14). It has been reported that every 10% increase in TILs rate reduces the risk of recurrence or death by 13%, the risk of distant organ metastasis or death by 17%, and the risk of death by 16% (15). Although a clear cutoff value for TILs related to the prognosis is not reported in the WHO Classification of Breast Tumors, a threshold of 50-60% is generally accepted (1). In our study, no relationship was found between TILs and survival rates, but the rate of TILs was significantly higher in HER2 and triple negative subgroups. In addition, the tumor stage was higher in tumors with lower TILs, which can be interpreted as a poorer clinical course for tumors showing low TILs.

Another parameter that has not been widely investigated in breast cancer, but has been found to be an independent prognostic and predictive marker in various malignancies such as colorectal and pancreoticobiliary system cancers, is the feature of the stroma (16,17). It has been reported that stromal reaction in breast tumors is associated with the rate of lymph node and distant organ metastasis and has an effect on prognosis (18). In their study of triple-negative breast cancers, Zakhartseva and Yanovytska determined the tumor stroma ratio to be an independent prognostic parameter associated with both disease-free survival and total survival periods (19). Roeke et al. have shown that a high tumor/stroma ratio is an independent prognostic parameter for total survival rate, distant metastasis, and recurrence-free survival rates. The clinical course is worse in cases with a high tumor stroma ratio. At the same time, this ratio is associated with advanced age and larger tumor size (20). In our study, the stroma was evaluated independently in the intratumoral and peritumoral areas. Contrary to other studies, the ratio of intratumoral stroma and tumor grade, size and ki-67 proliferation index were found to be inversely proportional. This is thought to be due to the difference of evaluation. In addition to the intensity of the stromal reaction, different histomorphological features of the stroma such as desmoplastic, hyalinized, or myxoid, are also known to have an effect on the prognosis. Yanai et al. found fibrotic stroma to be associated with a higher venous invasion rate and tumor grade. In addition, a fibrotic stroma is associated with a worse recurrence-free and total survival rate in triple-negative tumors (21). Similarly in our study, we found that tumors with a desmoplastic stroma were of higher grade, had higher Ki-67 proliferation indices and were more frequent in triple negative cases. Tumor budding is another important feature of the tumor microenvironment. It has been evaluated since it was first described in 1954 and found to be strongly related to the prognosis. In contrast to pancreatic, lung, and gastrointestinal system tumors, research on tumor budding is limited in breast cancer (22–25). However, studies show that tumor budding is an important parameter associated with lymphovascular invasion, lymph node metastasis and survival in this cancer (25,26). The study of Kumarguru et al. has shown a significant correlation between tumor budding and lymphovascular invasion, lymph node metastasis, necrosis, and tumor stage (27). Liang et al. have determined that the tumor size was larger, lymph node metastasis was more common, and the overall survival was shorter in cases with higher numbers of budding (28). Evaluation of tumor budding with different methods leads to different results. The evaluation in our study was done in a way similar to Renuka et al.’s study, in two separate areas as intratumoral and peritumoral. Intratumoral tumor budding was associated with lymph node metastasis, while peritumoral tumor budding was associated with lymphovascular invasion (26).

The relationship between tumor microenvironment and various prognostic parameters was studied in our study, but a relationship with recurrence-free and total survival rate was not detected. Despite the high number of cases included, this may suggest the need for a larger studies on breast cancer. The relatively low number of HER2 positive and triple negative cases is one of the limitations of this study. The prolongation of the total survival periods and the decrease in recurrence rates in breast cancer with the help of various treatment methods, also brings up the need of conducting surveillance studies with longer follow-up periods. In our study, the cases were evaluated over a 5-year follow-up period. This is one of the limitations of this study and longer follow-up periods may change the results. The tumor microenvironment is an important prognostic and predictive parameter. The fact that the evaluation can be made from H&E stained slides and that it does not cause additional costs and time loss makes it possible to easily evaluate these parameters worldwide. The relatively limited number of studies in this area in breast cancer prevents routine evaluation and the inclusion of these parameters in reporting formats. However, we believe that it will be possible to set a worldwide standard as the number of studies in this area increases.

## Funding

There were no external sources of funding for the present study.

## Ethics Committee Approval

Approval for the research, dated 25 November 2020 and numbered 2020-21/10, was obtained from the local Clinical Research Ethics Committee.

## Conflict of Interest

The authors declare that they have no conflict of interest.
